# Iron accumulation in the oculomotor nerve of the progressive supranuclear palsy brain

**DOI:** 10.1038/s41598-021-82469-w

**Published:** 2021-02-03

**Authors:** Hansol Lee, Myung Jun Lee, Eun-Joo Kim, Gi Yeong Huh, Jae-Hyeok Lee, HyungJoon Cho

**Affiliations:** 1 Department of Biomedical Engineering, Ulsan National Institute of Science and Technology, 50, UNIST-Gil, Eonyang-eup, Ulju-gun, Ulsan, South Korea; 2 Department of Neurology, Pusan National University Hospital, Pusan National University School of Medicine and Biomedical Research Institute, Busan, South Korea; 3 Department of Forensic Medicine, Pusan National University School of Medicine, Yangsan, South Korea; 4 Department of Neurology, Research Institute for Convergence of Biomedical Science and Technology, Pusan National University Yangsan Hospital, 20, Geumo-ro, Mulgeum-eup, Yangsan-si, Gyeongsangnam-do South Korea

**Keywords:** Biomarkers, Neurology, Biomarkers, Neurological disorders

## Abstract

Abnormal iron accumulation around the substantia nigra (SN) is a diagnostic indicator of Parkinsonism. This study aimed to identify iron-related microarchitectural changes around the SN of brains with progressive supranuclear palsy (PSP) via postmortem validations and in vivo magnetic resonance imaging (MRI). 7 T high-resolution MRI was applied to two postmortem brain tissues, from one normal brain and one PSP brain. Histopathological examinations were performed to demonstrate the molecular origin of the high-resolution postmortem MRI findings, by using ferric iron staining, myelin staining, and two-dimensional laser ablation-inductively coupled plasma-mass spectrometry (LA-ICP-MS) imaging. In vivo iron-related MRI was performed on five healthy controls, five patients with Parkinson’s disease (PD), and five patients with PSP. In the postmortem examination, excessive iron deposition along the myelinated fiber at the anterior SN and third cranial nerve (oculomotor nerve) fascicles of the PSP brain was verified by LA-ICP-MS. This region corresponded to those with high *R*_2_^*^ values and positive susceptibility from quantitative susceptibility mapping (QSM), but was less sensitive in Perls’ Prussian blue staining. In in vivo susceptibility-weighted imaging, hypointense pixels were observed in the region between the SN and red nucleus (RN) in patients with PSP, but not in healthy controls and patients with PD. *R*_2_^*^ and QSM values of such region were significantly higher in patients with PSP compared to those in healthy controls and patients with PD as well (vs. healthy control: *p* = 0.008; vs. PD: *p* = 0.008). Thus, excessive iron accumulation along the myelinated fibers at the anterior SN and oculomotor nerve fascicles may be a pathological characteristic and crucial MR biomarker in a brain with PSP.

## Introduction

The common pathological characteristics of Parkinsonism are elevated iron deposition with neuronal degeneration in the substantia nigra (SN)^1,2^. Several studies have investigated the neurochemistry of iron-containing molecules involved in the pathology of Parkinsonism^3,4^. Labile iron in the brain is associated with the generation of hydroxyl radicals and reactive oxygen species (ROS), leading to oxidative stress and cellular damage^5,6^. Specifically, high iron accumulation in the SN associated with disease progression results in detrimental damage to neuromelanin-containing dopaminergic neurons^7,8^. Iron accumulation in the SN can be visualized and monitored using in vivo magnetic resonance imaging (MRI) using iron-sensitive sequences. *R*_2_^*^ (1/*T*_2_^*^) map and quantitative susceptibility mapping (QSM) have been applied to quantify the iron concentration within the SN of patients of Parkinson’s disease (PD) and other Parkinsonian syndromes, including progressive supranuclear palsy (PSP), and compared to those in healthy controls^9^.

PSP is a degenerative parkinsonism characterized by hyperphosphorylated tau protein pathology and neuronal cell loss in cortical and subcortical structures, including the SN and midbrain structures, globus pallidus, and subthalamic nucleus^10,11^. Supranuclear vertical gaze palsy with significant midbrain atrophy has been recognized as a cardinal feature of PSP^12,13^. Iron accumulation within the PSP brain also serves as a potential biomarker in in vivo MRI studies, and it has the ability to help distinguish patients with PSP from normal controls^9,14,15^. Significant increases in iron-related signals have been found in the SN, red nucleus (RN), and globus pallidus of patients with PSP^9^. In postmortem MRI studies with pathological validation, the microstructural destruction of the borders and internal architecture of the SN is far greater in PSP than that in PD^16,17^. In FLASH MR images, the hypointense pixels near the boundary of the RN that adjoin the hypointense pixels of the SN resulted in less delineation between the structures of the PSP midbrain^16^. However, the exact underlying pathology of these alterations is unknown.

This work focuses on verifying the histological origin of increased MR susceptibility contrast between the SN and RN in the PSP midbrain and on ascertaining its utility as an in vivo diagnostic marker for PSP brains, which can differentiate these patients from healthy controls and patients with PD. 7 T high-resolution postmortem MRI of PSP and normal brains along with mutually independent iron characterization techniques including histopathology and mass spectrometry were collectively investigated. 3 T in vivo iron-related MRIs of the brains of healthy controls, PD, and PSP were compared.

## Results

### Postmortem study

**Figure 1:**
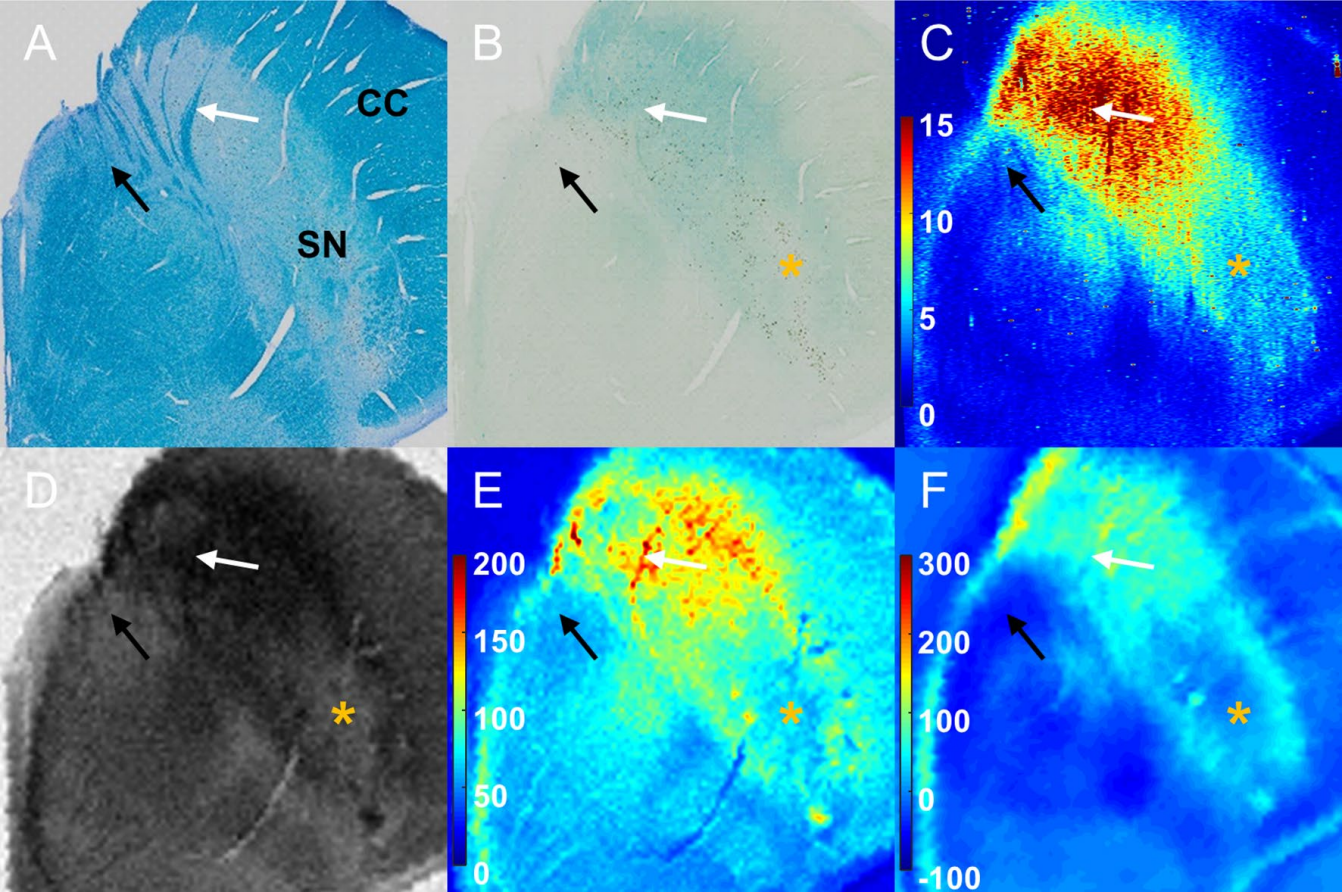
Histopathology and LA-ICP-MS with multimodal MRI on the postmortem SN of an 86-year-old normal female. (**A**): Luxol fast blue staining; (**B**): Perls’ Prussian blue staining; (**C**): ^56^Fe/^13^C intensity from LA-ICP-MS imaging (a.u.); (**D**): SWI; (**E**): *R*_2_^*^ map (1/s); (**F**): QSM (ppb); White and black arrows indicate myelinated fibers at anterior SN and oculomotor nerve. Orange asterisk shows the structure of nigrosome-1. CC = crus cerebri; LA-ICP-MS = laser ablation-inductively coupled plasma-mass spectrometry; QSM = quantitative susceptibility mapping; SN = substantia nigra; SWI = susceptibility-weighted imaging. Figures were generated from MATLAB (version R2016a, MathWorks, Natick, MA, USA).

**Figure 2:**
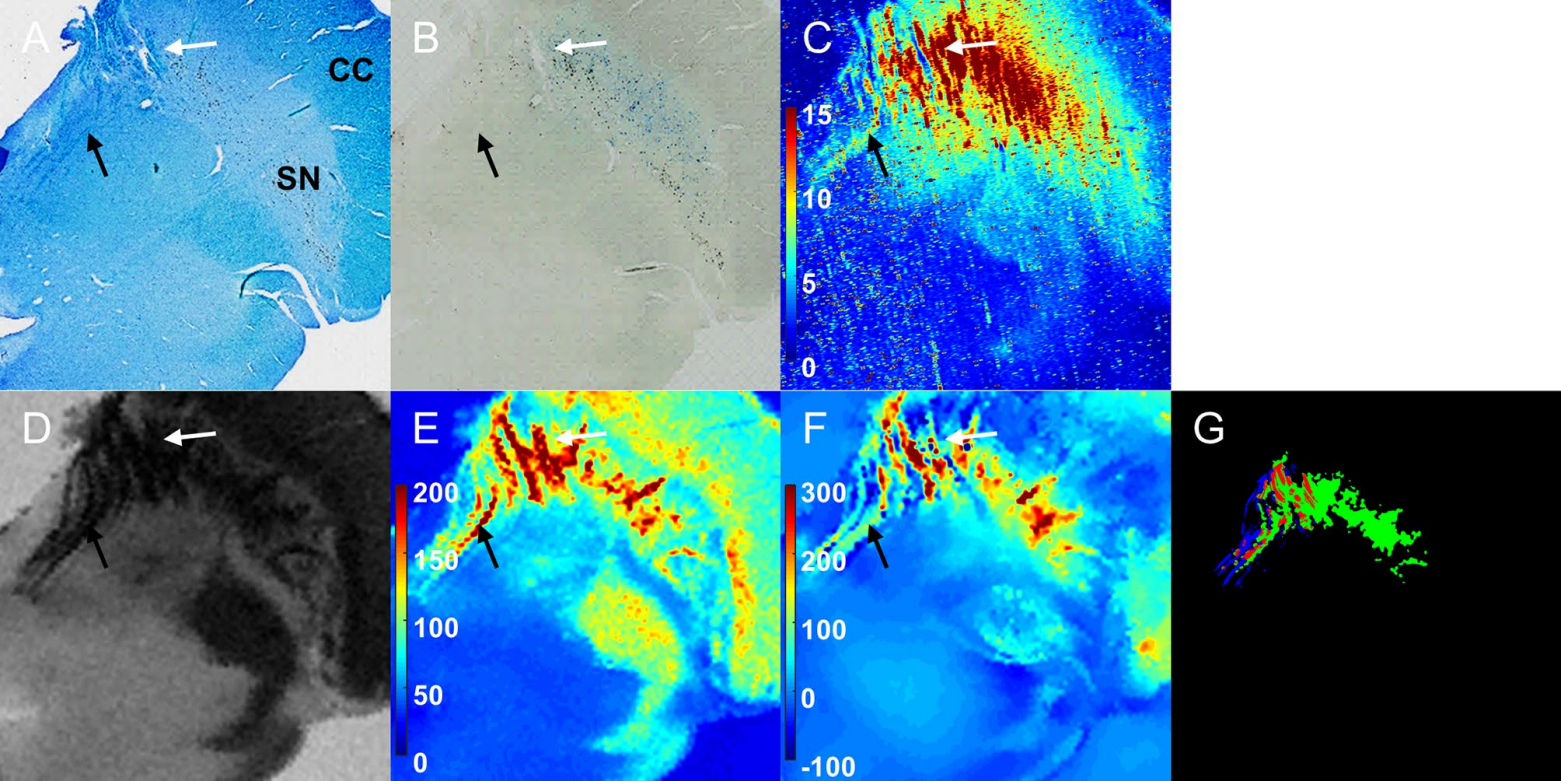
Histopathology and LA-ICP-MS with multimodal MRI on the postmortem SN of a 67-year-old male with PSP. (**A**): Luxol fast blue staining; (**B**): Perls’ Prussian blue staining; (**C**): ^56^Fe/^13^C intensity from LA-ICP-MS imaging (a.u.); (**D**): SWI; (**E**): R_2_^*^ map (1/s); (**F**): QSM (ppb). (**G**) The overlap (red) between myelinated fiber (blue) and hypointense SWI area (green). White and black arrows indicate myelinated fibers at anterior SN and oculomotor nerve. CC = crus cerebri; LA-ICP-MS = laser ablation-inductively coupled plasma-mass spectrometry; PSP = progressive supranuclear palsy; QSM = quantitative susceptibility mapping; SN = substantia nigra; SWI = susceptibility-weighted imaging. Figures were generated from MATLAB (version R2016a, MathWorks, Natick, MA, USA).

The co-registered results of multimodal high-resolution MRI, histopathology, and the two-dimensional image of iron distribution from laser ablation-inductively coupled plasma-mass spectrometry (LA-ICP-MS) of the postmortem normal and PSP brains are presented in Figs. 1 and 2, respectively.

Overall, the myelinated fibers at the anterior SN and third cranial nerve (oculomotor nerve) fascicles were observed in Luxol fast blue staining (white arrow for myelinated fibers at the anterior SN and black arrow for the oculomotor nerve fascicles in Figs. 1A and 2A). Ferric iron deposition was detected using Perls’ Prussian blue staining (Figs. 1B and 2B). The stained ferric iron was broadly distributed within the SN. The region of elevated intensity for ^56^Fe/^13^C in LA-ICP-MS imaging (Figs. 1C and 2C) corresponded with the areas of the stained ferric iron deposits (blue blush in Perls’ Prussian blue staining)^18^.

In the case of the normal brain, the area of deposited iron corresponded to the hypointense pixels in susceptibility-weighted imaging (SWI) (Fig. 1D), and large *R*_2_^*^ values were correspondingly observed in the same area (Fig. 1E). In the QSM (Fig. 1F), ferric iron deposition was shown as paramagnetic molecules. The structure of the so-called nigrosome-1 (the area of the orange asterisk) was detected as a hyperintense area in the SWI. This area was also described as an oval-shaped area with lower *R*_2_^*^ values and susceptibility compared to those of surrounding tissues. Nigrosome-1 had lightly stained iron distribution in Perls’ Prussian blue staining and a low intensity of iron (^56^Fe/^13^C). The *R*_2_^*^ map and QSM further showed linear myelinated fibers with moderately high values distributed in the anterior SN (white arrow in each image). No iron accumulation along the oculomotor nerve fascicles (black arrow) was observed on iron-related MRI or ^56^Fe/^13^C from LA-ICP-MS.

In the case of PSP, the volume atrophy of the midbrain was apparent compared to that in normal SN. Similar to a normal SN, iron deposition was identified as large *R*_2_^*^ values in Fig. 2E. In particular, the region with both large values in the *R*_2_^*^ map and positive susceptibility values in the QSM (Fig. 2F) included the oculomotor nerve (black arrow) fascicles and myelinated fibers in the anterior SN (white arrow) identified from Luxol fast blue staining. The ^56^Fe/^13^C intensity from LA-ICP-MS also showed a significant iron signal in the area of myelinated fiber at the anterior SN and oculomotor nerve fascicles, directly indicating iron accumulation along the oculomotor nerve. The spatially overlapped region (red) between segmented myelinated fibers from Luxol fast blue staining (blue) and hypointense regions from SWI (green) is shown in Fig. 2G. On the other hand, such iron distribution along the myelinated fibers in the PSP brain was not sensitively stained in the Perls’ Prussian blue staining (Fig. 2B).

Considering the age and gender differences between the normal control and PSP brains, the additional results of Luxol fast blue, Perls’ Prussian blue staining, *R*_2_^*^ map, and QSM of the postmortem midbrain of a 60-year-old normal male and a 70-year-old normal female are presented in Supplementary Fig. 1. The myelinated fibers of the oculomotor nerve detected in Luxol fast blue staining were not distinctively shown in the *R*_2_^*^ map and QSM as indicated by red arrows. The mild iron concentration along myelinated fibers in the two normal control cases was not enough to overwhelm the effect of diamagnetic myelinated fibers in QSM, which was clearly observed in 67-year-old male PSP brains.

### In vivo MRI

**Figure 3:**
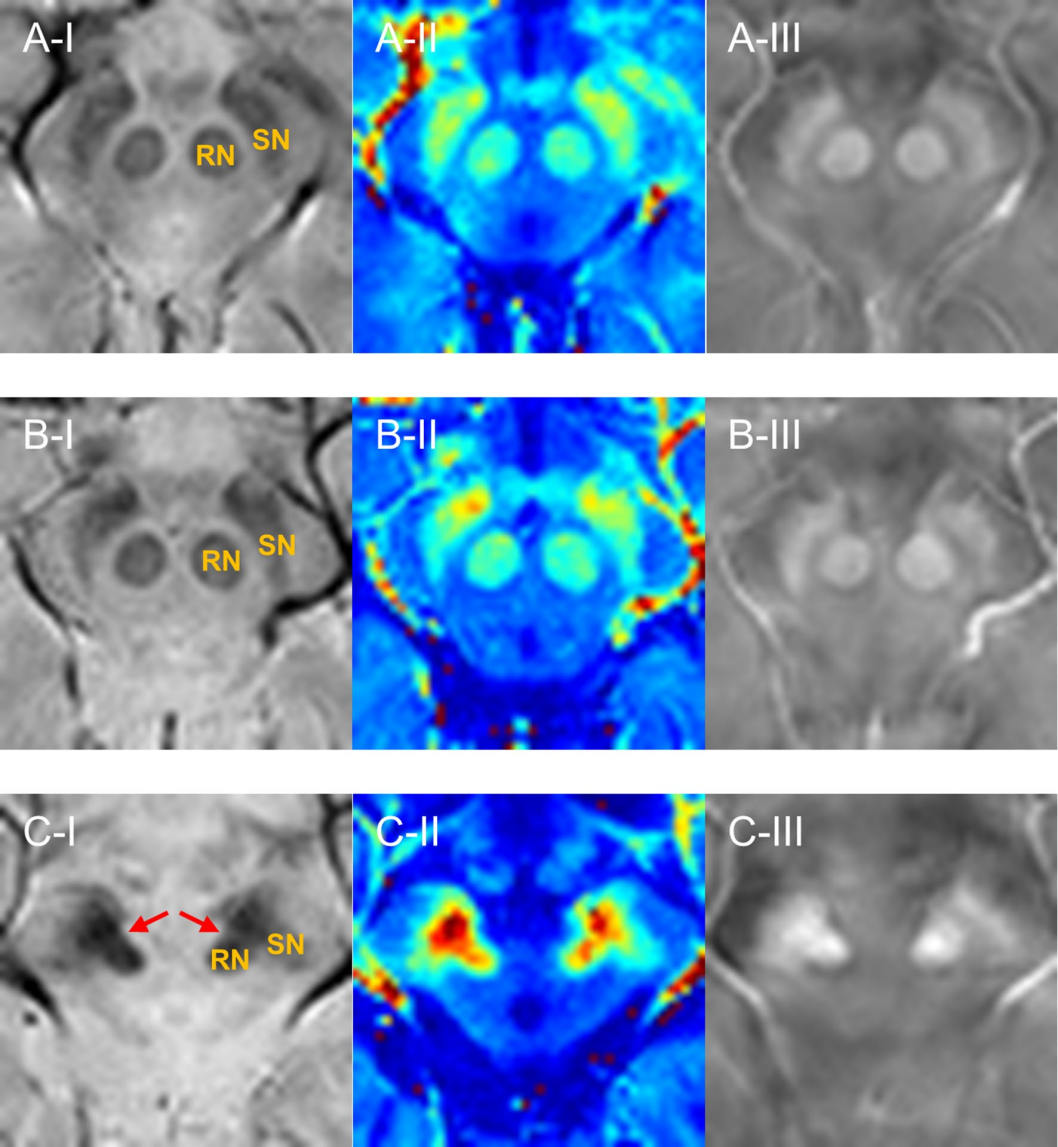
The representative MRI showing the SN and RN. (**A**) Representative case (61F) of the healthy control group. (**B**) Representative case (60M) of the PD group. (**C**) Representative case (66M) of the PSP group. The red arrows in (**C**-**I**) show the hypointensity in the region between SN and RN. (I): SWI; (II): *R*_2_^*^ map; (III): QSM. PD = Parkinson’s disease; PSP = progressive supranuclear palsy; QSM = quantitative susceptibility mapping; SN = substantia nigra; SWI = susceptibility-weighted imaging; RN = red nucleus. Figures were generated from MATLAB (version R2016a, MathWorks, Natick, MA, USA).

For in vivo MRI, representative MR images of the SWI, *R*_2_^*^ map, and QSM showing rostral SN in healthy control, PD, and PSP groups are presented in Fig. 3. The values of *R*_2_^*^ map and QSM in SN were highest in patients with PSP, followed by patients with PD and then healthy controls. Marked atrophy of the midbrain was observed in all patients with PSP compared to those of healthy controls and patients with PD in the same field of view. In the SWI, a clear hyperintense boundary was identified in the region between the hypointense SN and the hypointense RN in healthy controls and patients with PD (Fig. 3A-I, B-I). However, in the case of PSP, the hypointense area was shown at the areas bridging the SN and RN as an atypical connection between two tissues with blurred boundaries (Fig. 3C-I). This connection was also identified in the *R*_2_^*^ map and QSM of patients with PSP (Fig. 3C-II,III).

**Figure 4:**
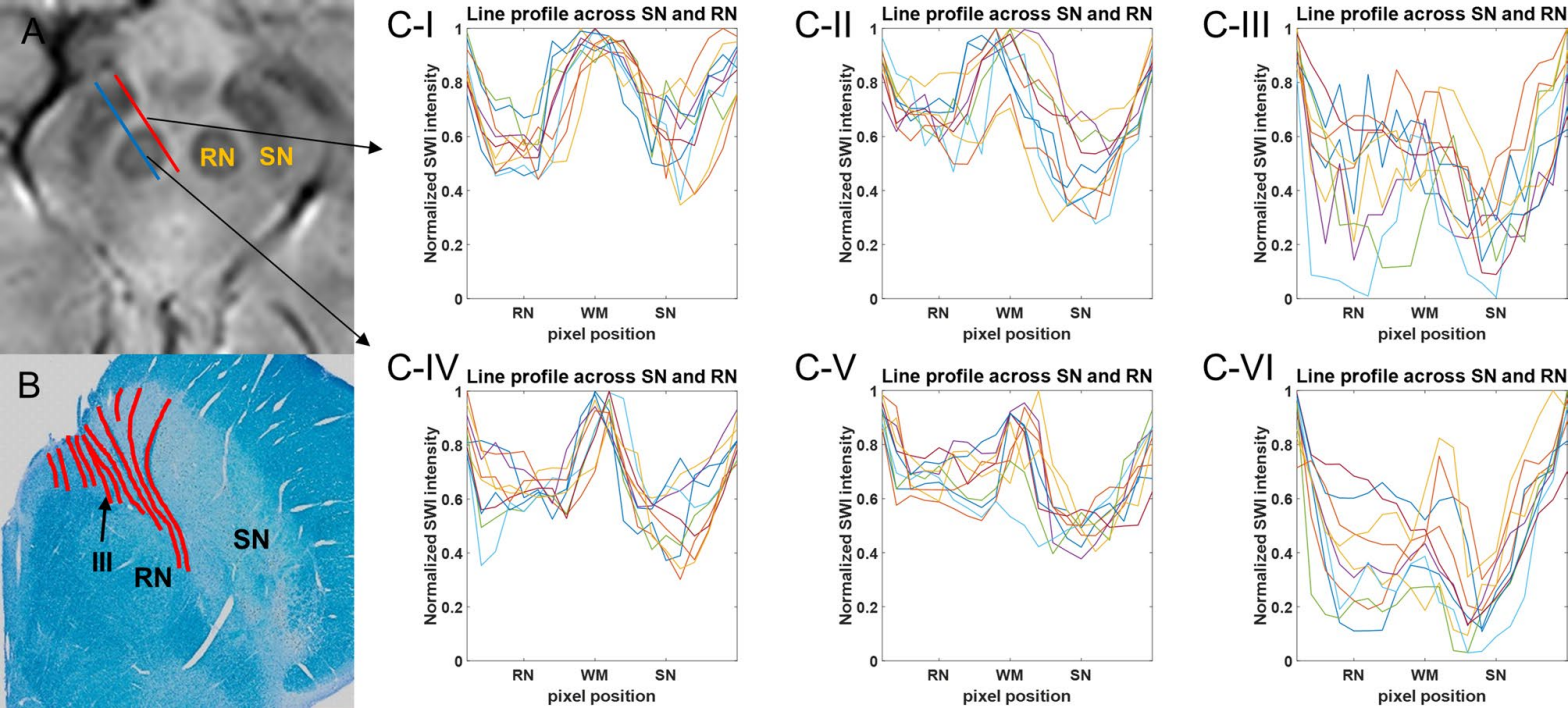
The line profile of normalized SWI intensity across the SN and RN. (**A**): SWI (**B**): Luxol fast blue staining with red lines for myelin distribution; (**C-I**), (**C-IV**): The line profile of normalized SWI intensity of healthy controls; (**C-II**), (**C-V**): The line profile of normalized SWI intensity of patients with PD; (**C-III**), (**C-VI**): The line profile of normalized SWI intensity of patients with PSP. The line profiles of (**C-I**–**C-III**) were delineated along the red line in SWI. The line profiles of (**C-IV**–**C-VI**) were delineated along the blue line in SWI. PD = Parkinson’s disease; PSP = progressive supranuclear palsy; SN = substantia nigra; RN = red nucleus; WM = white matter between SN and RN. (**A**,**B**) were generated from MATLAB (version R2016a, MathWorks, Natick, MA, USA).

In Fig. 4, the respective line profiles of normalized SWI intensities are presented across the SN and RN along the two separate lines of the three groups, as shown in Fig. 4A. To visualize the selectivity of such lines to myelinated white matter, the myelin of the oculomotor nerve fascicles passing by the RN and the myelin in the anterior SN were connected by red lines in Luxol fast blue staining, as shown in Fig. 4B. Ten line profiles of healthy controls along both red and blue lines in the SWI (Fig. 4C-I,IV) showed hyperintensity in the white matter region (WM in the x-axis) bridging the SN and RN. Hyperintense areas were also maintained in the white matter region between the SN and RN in patients with PD (Fig. 4C-II,V). Conversely, for patients with PSP, the SN, RN, and white matter region between the two tissues were hypointense, which resulted in blurred structural boundaries (Fig. 4C-III,VI). The line profiles for *R*_2_^*^ and QSM values along the blue line in SWI are presented in Supplementary Fig. 2. The line profiles of *R*_2_^*^ and QSM showed a concave pattern in the region between the SN and RN in both healthy controls and patients with PD, but such trend was significantly decreased in patients with PSP.

The mean and standard deviation of *R*_2_^*^ and QSM values in the region bridging the SN and RN were compared among the three groups (Table 1). *R*_2_^*^ and QSM values of PSP were significantly higher than those of the other two groups (*R*_2_^*^: *p* = 0.008 with healthy controls, *p* = 0.008 with PD; QSM: *p* = 0.008 with healthy controls, *p* = 0.008 with PD). However, the differences in *R*_2_^*^ and QSM values between healthy controls and PD groups were negligible (*p* = 0.421 and *p* = 1, respectively) in the region bridging the SN and RN. For the datasets with separated left and right sides of the brain, *R*_2_^*^ and QSM values of PSP were more significantly distinguishable from those of other two groups*,* as demonstrated by the *p*-values in parentheses.

**Table 1:** Comparison of R_2_^*^ and QSM in the region between SN and RN among PD, PSP and healthy control groups.

	Control	PD	PSP	Kruskal–Wallis		post-hoc (*p*)		
				${\chi }^{2}$	*p*	Control vs. PD	Control vs. PSP	PD vs. PSP
R_2_* values (s^−1^)	33.7 ± 1.6	37.3 ± 5.6	54.9 ± 8.0	9.8	0.008*	0.421 (0.473)	0.008* (< 0.001**)	0.008* (0.002**)
QSM (ppb)	31.2 ± 15.4	34.1 ± 11.9	116.2 ± 23.7	9.4	0.009*	1 (0.910)	0.008* (< 0.001*)	0.008* (< 0.001**)

Data are shown as mean ± standard deviation.

Post-hoc (*p*) = *p*-values from post-hoc analysis of Mann–Whitney U test.

Control = healthy control; PD = Parkinson’s disease; PSP = progressive supranuclear palsy; SN = substantia nigra; RN = red nucleus.

*p < 0.05 (For post-hoc analysis, *p < 0.05/3 and **p < 0.005/3 after Bonferroni correction).

## Discussion

The main finding in this study is that iron deposition along the myelinated fibers at the anterior SN and oculomotor nerve fascicles in the brain of patients with PSP can be visualized using multiple independent approaches, such as 7 T postmortem MRI, histological analysis, mass spectrometry, and in vivo 3 T MRI. Our postmortem examinations showed that the increased MR susceptibility contrast between the SN and RN identified in patients with PSP is likely to originate from excessive iron deposition along the myelinated nerves between these two structures. To our knowledge, this is the first report demonstrating a higher level of iron deposition along the myelinated fibers at the anterior SN and oculomotor nerve fascicles in the vicinity of the SN and RN of patients with PSP compared to those of patients with PD and healthy controls by using *R*_2_^*^, QSM, and LA-ICP-MS.

Atrophy of the midbrain, which is a recognized characteristic in patients with PSP, may shorten the gap between the SN and RN. The shortened gap between the SN and RN probably causes an atypical connection between the two structures with blurred boundaries in low-resolution on in vivo MRI. Regardless of the volume loss in PSP brain, the fibers of oculomotor nerve would still present between the SN and RN. In our postmortem examinations of the PSP midbrain, Luxol fast blue staining showed the distribution of myelinated fibers at the anterior SN and the oculomotor nerve fascicles, which had a considerably high iron (^56^Fe/^13^C) signal intensity in the LA-ICP-MS image. The corresponding area had large *R*_2_^*^ values and positive susceptibility values in the QSM. The spatial overlap between myelinated fibers from Luxol fast blue staining and the hypointense region in SWI demonstrated that the myelinated fibers with high iron concentration were non-invasively observed in the iron-related MRI. However, Perls’ Prussian blue staining, which is the conventional method used to determine the distribution of ferric iron within brain tissues, was less sensitive for staining myelin-associated iron.

It is still unclear why there is a high concentration of iron deposits along the myelinated fibers of the oculomotor nerve in association with PSP progression. There are several possible explanations for this abnormal iron deposition. (1) In human studies, high levels of iron have been reported to co-localize with hyperphosphorylated tau aggregates^19^. Tau-containing globose neurofibrillary tangles are prevalent in the PSP midbrain, including in the oculomotor nerve complex^20^. (2) Anatomically, the fibers of the oculomotor nerve fascicles from the nucleus pass by the RN and SN, and these two structures contain a high level of iron concentration^17^. Excessive iron accumulation within both the SN and RN can also cause abnormal iron deposition along the nearby myelinated fiber. (3) The vulnerability of myelinated fibers and oligodendrocytes to oxidative stress may be further accelerated by their high iron environment as myelination and axon maturation require iron consumption^21,22^. The dysfunction of the iron homeostasis mechanism in myelinated fibers, such as impaired iron transportation of the iron transport tract and imperfect iron excretion from the neurons, can cause high iron deposition along the associated myelinated fibers^23,24^. The increased iron level along the myelinated fiber of the oculomotor nerve is likely to result in neuronal damage with disease progression^7^.

Although white matter, including myelinated fibers, is originally considered the main diamagnetic source in the brain due to its heavy phospholipid component, a high level of iron-containing molecules is also stored in myelin and oligodendrocytes as they have high iron requirements^25^. As demonstrated in the present study, iron deposition along the myelinated fiber around the SN can be a specific endogenous iron cluster, because the overloads of iron deposition along myelinated fibers overwhelm diamagnetism and induce paramagnetism as a myelin-iron complex^25,26^. Moreover, various forms of endogenous iron clusters are distributed within the SN, including the neuromelanin-iron complex, reactive ferric irons in neurons and glial cells, pathological hallmarks of disorders apart from iron such as α-synuclein or tau, and the heme iron in microvessels crossing the SN^27–30^. All these endogenous iron clusters within the SN need to be interpreted with care in in vivo iron-related MR contrast.

This study had several limitations. First, the postmortem sample size was small. However, a previous postmortem study on the SN of normal control and PSP brains showed consistent hypointensity between the SN and RN only in the PSP brain^16^. Second, we have not presented the corresponding images from postmortem PD midbrains for direct comparison between PD and PSP midbrain tissues. In PD, the most severely affected regions are reported to be nigrosomes, containing most dopaminergic neurons, at the posterior SN^31^. The evaluation of the presence of nigrosome-1 in the posterior SN (rather than the anterior SN in the analysis of PD brain) in MRI has been utilized as a promising biomarker for PD diagnosis. Third, the disease severity (H-Y stage) was different between patients with PD and PSP, which may influence MR contrast. PSP is known to progress more rapidly than PD, and it is difficult to match disease duration and H-Y stage together between the two groups^32^. Further investigation should be pursued on a larger number of subjects with no significant differences in age, sex, and disease severity in each group. Histopathological validation is also recommended for postmortem PD and PSP brains with a large sample in future studies.

In conclusion, the current study has demonstrated excessive iron deposition along the myelinated fiber at the anterior SN and the third cranial nerve (oculomotor nerve) in the PSP brain, applied this knowledge to understand the in vivo iron-related MR contrast seen in patients with PSP, and compared it to those of healthy controls and patients with PD. Consequently, it was found that the connection between the SN and RN in in vivo SWI, *R*_2_^*^ map, and QSM in patients with PSP can be a useful MR biomarker in the differential in vivo diagnosis of patients with PSP from healthy controls, patients with PD, and other patients with atypical Parkinsonian syndrome.

## Methods

This study was approved by the Pusan National University Yangsan Hospital and Ulsan National University of Science and Technology institutional review board. All procedures, including the in vivo MRI, postmortem MRI, mass spectrometry, and histopathological analysis were conducted according to the guidelines of the Helsinki Declaration. The images were processed using in-house developed MATLAB codes (version R2016a, MathWorks, Natick, MA, USA).

### Postmortem MRI and histopathological analysis

Midbrain specimens of an 86-year-old female without any neurodegenerative disease were acquired from the Pusan National University Anatomical Donation Program. A diseased midbrain tissue, which was characterized by severe midbrain atrophy, frontotemporal lobar degeneration, tau pathology, and depigmentation in the SN, from a 67-year-old male diagnosed with PSP was obtained from the Pusan National University Hospital Brain Bank. The formalin-fixed midbrain samples were stored in a 4 °C refrigerator for more than 2 years for sufficient stabilization of MR properties^33,34^. Formalin fixation redistributes iron within the tissues^35^. Although it may alter the staining intensity and the absolute transverse relaxometry values (*R*_2_^*^) in postmortem MR images, the contrast of postmortem SWI, *R*_2_^*^, and QSM within brain tissues were maintained from in vivo MRI^35,36^.

Two tissues were placed in 50 mL syringes separately after removing air bubbles because bubbles around the tissue surface cause susceptibility artifacts on MR images. High-resolution MR acquisitions were performed on tissues using 7 T preclinical MRI (Bruker, Karlsruhe, Germany) at Ulsan National University of Science and Technology to validate the origin of the in vivo MRI contrast. SWI, *R*_2_^*^ map, and QSM were used to evaluate iron and myelin contents in 2D multiple gradient echo sequence acquired using the following parameters: repetition time (TR) = 2000 ms, echo time (TE) = 3.3–81.2 ms (20 echoes with $\Delta$TE = 4.1 ms), flip angle = 30°, field of view = 35 × 35 mm, matrix size = 256 × 256, slice thickness = 500 μm, and number of slices = 20. The slice geometry was perpendicular to the main magnetic field. SWI was acquired using magnitude and phase images of TE = 15.6 ms. The *R*_2_^*^ map was obtained from the magnitude image by mono-exponential fitting of the *T*_2_^*^ transverse relaxation curve on each voxel. QSM was reconstructed from phase images of five tilted orientations using the Laplacian boundary value (LBV) algorithm for background field removal and calculation of susceptibility through multiple orientation sampling (COSMOS)^37,38^. *T*_*1*_-weighted images were also acquired with 2D RARE sequence for co-registration with histological analysis using the following parameters: TR = 800 ms, TE = 8 ms, flip angle = 30°, field of view = 35 × 35 mm, matrix size = 256 × 256, slice thickness = 500 μm, and number of slices = 20.

After the MR scan, tissue samples were subjected to histopathological analysis, which is a gold standard to demonstrate the effect of underlying elements on the corresponding MR images. For the tissue cryoprotection, to minimize osmotic stress and ice formation during cooling, tissues were sequentially embedded in 10%, 20%, and 30% sucrose in Phosphate-buffered saline solution until they sank. Thin slides of 50 μm thickness were generated using a cryostat (CM1950, Leica Biosystems, Nussloch, Germany). Ten sectioned slides (thickness 50 μm) were prepared from one corresponding MR image (thickness 500 μm). Of ten sections, three adjacent slides were used serially for Perls’ Prussian blue staining, Luxol fast blue staining, and LA-ICP-MS, respectively.

Perls’ Prussian blue staining was performed for detecting ferric iron distribution. For Perls’ Prussian blue staining, the slides were incubated in a 1:1 mixed solution of 20% HCl and 20% potassium ferrocyanide for 30 min. Luxol fast blue staining was performed to identify the distribution of myelinated fibers by soaking the tissues in 0.1% filtered Luxol fast blue solution at 65 °C in an oven overnight and counterstained with 0.1% cresyl violet acetate solution. The histological slides were imaged using Virtual Microscope (Olympus Optical Co. Ltd, Tokyo, Japan).

LA-ICP-MS was conducted to detect all molecular forms of iron within brain tissues on the slide that was neither stained with Perls’ Prussian blue nor Luxol fast blue. Two-dimensional images of ^56^Fe and ^13^C intensity were obtained by line scan using a quadrupole ICP-MS device, iCAP TQ (ThermoFisher Scientific, Bremen, Germany) with a femtosecond laser (1030 nm) ablation system (J200, Applied Spectra, Inc, Fremont, CA, USA). For the comparison of iron concentration between tissues, ^56^Fe intensity was normalized by ^13^C intensity to compensate sample-to-sample variations in laser ablation measurements, as ^13^C is a suitable internal standard for quantitative elemental bio-imaging^39^.

For direct comparisons, postmortem MRI was co-registered with corresponding histological results and LA-ICP-MS images. Due to the different spatial resolutions among images, MR images and images of ^56^Fe and ^13^C intensity from LA-ICP-MS were up-sampled by bicubic interpolation before co-registration. The two-dimensional rigid transformation of rotation and translation was performed on up-sampled MR images and up-sampled images of ^56^Fe and ^13^C intensity from LA-ICP-MS to match the images of Luxol fast blue staining. The same transformation method was also used for the co-registration of the image of Perls’ Prussian blue staining to the image of Luxol fast blue staining.

### In vivo MRI

Five patients with PSP, along with five age-matched patients with PD and five age-matched healthy controls, were included in this study. The demographic features of all participants are summarized in Table 2. Patients were clinically diagnosed by a movement disorder neurologist in accordance with the established criteria for each disorder^40,41^. Although there was no significant difference in age and disease duration between patients with PD and those with PSP, the H-Y stage was significantly higher in patients with PSP (*p* = 0.008). All subjects provided informed consent and underwent 3 T in vivo MRI (Magnetom Skyra, Siemens, Erlangen, Germany) at Pusan National University Yangsan Hospital. SWI, *R*_2_^*^ map, and QSM were taken with a 2D gradient echo sequence using the following parameters: TR = 2030 ms, TE = 3.1–29.9 ms (6 echoes with $\Delta$TE = 4.8, 5.5, …, 5.5 ms), flip angle = 60°, field of view = 192 × 192 mm, matrix size = 192 × 192, slice thickness = 2 mm, and number of slices = 60. SWI was acquired using the magnitude and phase images of TE = 24.6 ms. The *R*_2_^*^ map was obtained using the same technique of postmortem MRI. QSM was reconstructed using MATLAB-based software, STI-Suite (version 3.0, University of California, Berkeley, CA, USA, https://people.eecs.berkeley.edu/~chunlei.liu/software.html). Among the 60 slices, the slice of the second level in the rostral direction showing the SN with a clear shape of the RN was consistently selected for each subject for the analysis.

**Table 2:** Demographic and clinical characteristics of PD, PSP, and healthy control groups.

	PD	PSP	Control
Subjects (M/F)	3/2	4/1	2/3
Age (years)	61.2 ± 3.7	64.2 ± 2.2	61.0 ± 2.7
Disease duration (years)	4.8 ± 3.1	3.4 ± 0.5	–
H-Y stage	2.0 ± 0.5	3.4 ± 0.5	–

Data are shown as mean ± standard deviation.

Control = healthy control; H-Y = Hoehn & Yahr; PD = Parkinson’s disease; PSP = progressive supranuclear palsy.

The line profile of normalized SWI intensity, *R*_2_^*^, and QSM across the SN and RN were plotted along the two different lines for each group (right and left sides of five subjects in each group). SWI intensity was normalized by the maximum intensity value of each line profile. The white matter region between the SN and RN was manually delineated in SWI by H.L. as shown in Supplementary Fig. 3. The quantitative values of *R*_2_^*^ and QSM within the region between the SN and RN using the same ROI from SWI were compared among the three groups using a Kruskal–Wallis H test. Bonferroni correction was performed for multiple comparisons with significance levels of 0.05/3 = 0.0166.

## Supplementary Information

Supplementary Information.

## Data Availability

The data of the postmortem examination and in vivo MRI with limited demographic and clinical information are available from the corresponding authors upon reasonable requests.

## Abbreviations

LA-ICP-MS: Laser ablation-inductively coupled plasma-mass spectrometry
MRI: Magnetic resonance imaging
PD: Parkinson’s disease
PSP: Progressive supranuclear palsy
QSM: Quantitative susceptibility mapping
RN: Red nucleus
ROS: Reactive oxygen species
SN: Substantia nigra
SWI: Susceptibility-weighted imaging
TE: Echo time
TR: Repetition time
WM: White matter

## Code availability

The MATLAB code used in this study is available from the corresponding authors upon reasonable requests.
