# Supplementary material for: Iron accumulation in the oculomotor nerve of the progressive supranuclear palsy brain
Source: Sci Rep. 2021 Feb 3;11:2950. doi: 10.1038/s41598-021-82469-w (PMC7859181; doi:10.1038/s41598-021-82469-w)
Supplement: Supplementary file 1 — Supplementary Information. [file 41598_2021_82469_MOESM1_ESM.pdf]

Supplementary Material

Iron accumulation in the oculomotor nerve of the progressive supranuclear palsy brain

Hansol Lee[1], Myung Jun Lee[2], Eun-Joo Kim[2], Gi Yeong Huh[3], Jae-Hyeok Lee[4,*], and HyungJoon Cho[1,*]

[1]Department of Biomedical Engineering, Ulsan National Institute of Science and Technology, Ulsan, South Korea

[2]Department of Neurology, Pusan National University Hospital, Pusan National University School of Medicine and Biomedical Research Institute, Busan, South Korea

[3]Department of Forensic Medicine, Pusan National University School of Medicine, Yangsan, South Korea

[4]Department of Neurology, Research Institute for Convergence of Biomedical Science and Technology, Pusan National University Yangsan Hospital, Yangsan, South Korea

Supplementary Figure

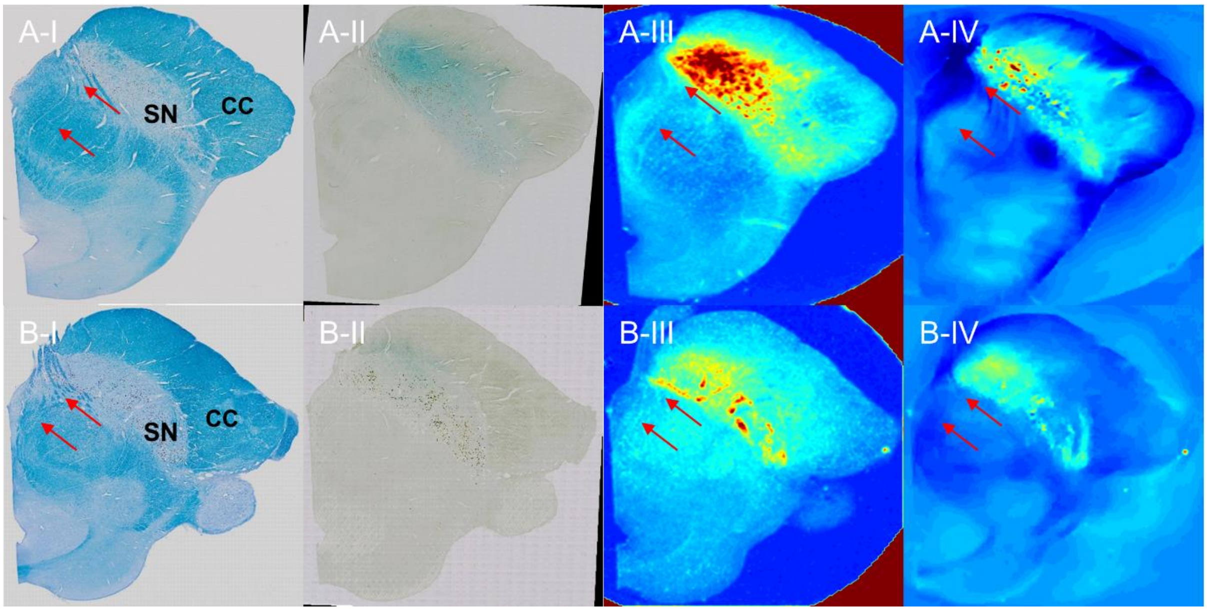

Supplementary Figure 1. **Histopathology and multimodal MRI on the postmortem SN of a 60-year-old normal male and 70-year-old normal female.** A: 60-year-old normal male. B: 70-year-old normal female. The red arrows indicate myelinated fibers of oculomotor nerve. (I): Luxol fast blue staining; (II): Perls' Prussian blue staining; (III): R_2^* map; (IV): QSM. QSM = quantitative susceptibility mapping; SN = substantia nigra. Figures were generated from MATLAB (version R2016a, MathWorks, Natick, MA, USA).

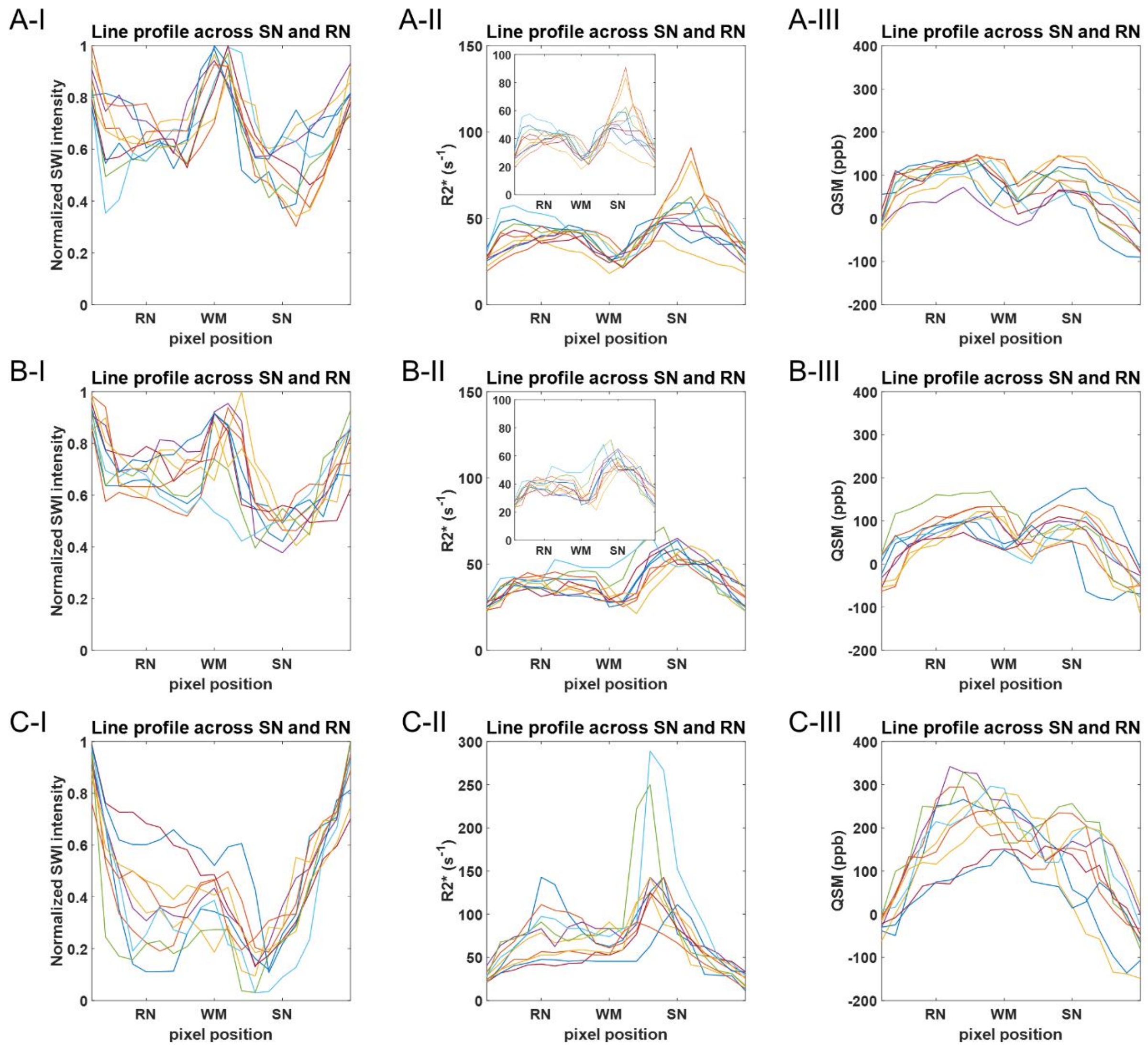

Supplementary Figure 2. **The line profiles across SN and RN.** A is for the representative case of a group of healthy controls. B is for the representative case of the PD group. C is for the representative case of the PSP group. The line profiles were delineated along the same blue line used in SWI (Figure 4A) of each subject. (I): The line profile of normalized SWI intensity; (II): The line profile of R_2^* (The same plot of A-II and B-II with magnified *y*-axis was exhibited as inset figure); (III): The line profile of QSM. PD = Parkinson's disease; PSP = Progressive supranuclear palsy; QSM = quantitative susceptibility mapping; RN = red nucleus; SN = substantia nigra; SWI = susceptibility-weighted imaging; WM = white matter region between SN and RN.

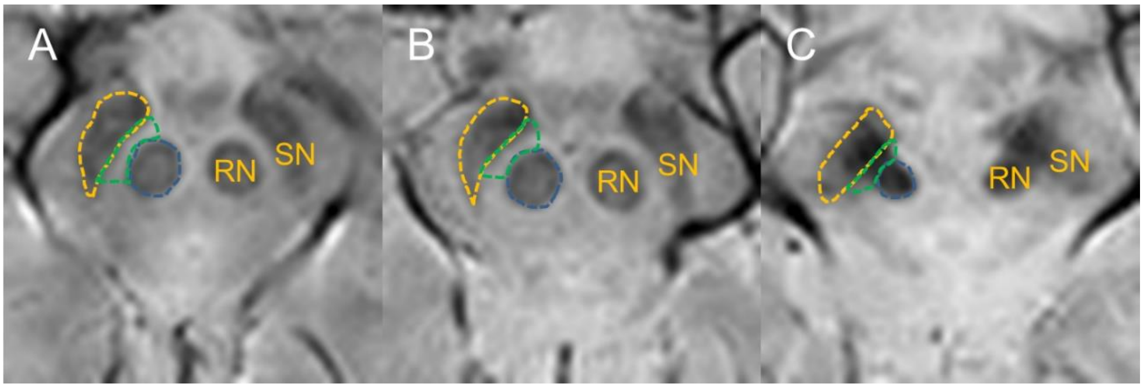

Supplementary Figure 3. **The delineation of ROI surrounding the region between SN and RN in SWI.** A: Representative case (61F) of the healthy control group. B: Representative case (60M) of the PD group. C: Representative case (66M) of the PSP group. The orange dotted line showed the boundary of SN. The blue dotted line showed the boundary of RN. The green dotted line showed the ROI surrounding the region between SN and RN. PD = Parkinson's disease; PSP = progressive supranuclear palsy; RN = red nucleus; ROI = region of interest; SN = substantia nigra; SWI = susceptibility-weighted imaging. Figures were generated from MATLAB (version R2016a, MathWorks, Natick, MA, USA).
